# The Effect of Fatigue on Throwing Performance in Handball Players: Systematic Review with Meta-Analysis

**DOI:** 10.3390/jfmk11020191

**Published:** 2026-05-12

**Authors:** Stelios Hadjisavvas, Irene-Chrysovalanto Themistocleous, Michalis A. Efstathiou, Elena Papamichael, Christina Michailidou, Manos Stefanakis

**Affiliations:** Physiotherapy Program, Department of Health Sciences, University of Nicosia, Nicosia 2414, Cyprus; hadjisavvas.s@unic.ac.cy (S.H.); themistocleous.i@unic.ac.cy (I.-C.T.); efstathiou.m@unic.ac.cy (M.A.E.); papamichael.el@unic.ac.cy (E.P.); michailidou.c@unic.ac.cy (C.M.)

**Keywords:** team handball, throwing velocity, throwing accuracy, jump shot, sports performance, biomechanics

## Abstract

**Background**: Acute fatigue is frequently experienced during handball training and match play and may impair throwing performance; however, findings across studies are inconsistent. This systematic review and meta-analysis examined the acute effects of fatigue on throwing velocity and accuracy in handball. **Methods**: PubMed, MEDLINE Complete, CINAHL, and Scopus were searched from inception to 24 January 2026, supplemented by Google Scholar and citation tracking. Eligible studies included handball players exposed to an acute fatigue protocol with throwing-performance outcomes. Random-effects meta-analyses were conducted using standardized mean differences (Hedges’ g), oriented so that negative values indicated worse performance under fatigue. **Results**: Τen studies met the inclusion criteria for qualitative synthesis. For quantitative synthesis, 10 comparisons contributed to the throwing-velocity meta-analysis and 6 comparisons contributed to the throwing-accuracy meta-analysis. Fatigue showed a small-to-moderate tendency to reduce throwing velocity (g = −0.31, 95% CI −0.65 to 0.03; I^2^ = 77.8%). For throwing accuracy, the pooled estimate suggested a possible decline under fatigue (g = −0.82, 95% CI −1.95 to 0.31), but heterogeneity was very high (I^2^ = 95.8%) and findings were sensitive to influential effects. **Conclusions**: Acute fatigue showed a small-to-moderate tendency to reduce throwing velocity in handball players, with more consistent impairments observed during jump-shot tasks and after localized upper-limb fatigue protocols. In contrast, no robust conclusion can be drawn for throwing accuracy/precision because heterogeneity was extremely high and studies used substantially different outcome definitions, including hit counts, success percentages, points-based scores, and spatial error. Therefore, accuracy findings should be interpreted with considerable caution.

## 1. Introduction

Τeam handball is a high-intensity intermittent team sport characterized by frequent accelerations and decelerations, repeated changes of direction, jumps, physical contact, and high-tempo technical actions across two halves [1,2]. Within this environment, the ability to execute effective shots on goal is central to offensive success, making throwing performance a key determinant of match outcome and player effectiveness [3]. In research and applied settings, throwing performance is most commonly quantified through measures of ball/throwing/release velocity and throwing or shooting accuracy, sometimes considered jointly as a speed–accuracy trade-off [3,4]. Because velocity and accuracy reflect distinct components of performance and may show different fatigue responses, they are best considered as separate outcomes [5].

From a biomechanical standpoint, high-velocity handball throwing depends on coordinated whole-body sequencing and efficient transfer of momentum through the kinetic chain, with technique-related kinematic parameters contributing meaningfully to ball velocity and accuracy [3]. Because this coordination relies on the integrity of neuromuscular function, any condition that perturbs force production, motor control, or intersegmental timing could plausibly impair throwing outcomes. Match play and high-intensity training in handball can impose substantial physiological and mechanical loads that accumulate over time, and match-induced impairments in physical performance have been reported in elite male players [2]. These observations support a practical concern: athletes often need to sustain throwing speed and precision under fatigue, precisely when decisive actions occur late in halves or during congested match schedules [1,2].

Fatigue is a multifaceted construct. In neuromuscular physiology, muscle fatigue is often described as an exercise-induced reduction in the capacity to produce maximal force, reflecting both peripheral mechanisms within the muscle and central mechanisms related to neural drive [6]. Contemporary frameworks additionally emphasize that observable performance decline (“performance fatigability”) is shaped by task demands and interacts with perceptions and regulatory processes (“perceived fatigability”) [5,7]. In the context of overhead throwing, fatigue may influence performance through reductions in force-generating capacity, altered motor control, and disrupted coordination; however, the specific mechanisms are likely to depend on the fatigue task, the muscles involved, and the characteristics of the athlete population [5,7]. Importantly, velocity and accuracy do not necessarily degrade in parallel; depending on the constraints of the task, athletes may adopt compensatory strategies that preserve one outcome at the expense of the other [4].

Understanding fatigue-related changes in throwing performance is also relevant to athlete health. Handball has a considerable shoulder injury and shoulder-problem burden, and systematic evidence suggests that shoulder issues are common and associated with modifiable factors, such as strength deficits/imbalances and training-load management [8]. In adolescent elite handball players, prospective monitoring has shown a notable prevalence of shoulder problems during the season, underscoring that shoulder-related limitations can emerge early and persist [9]. Although performance decrements under fatigue are not equivalent to injury risk, fatigue-induced alterations in force production and movement coordination are frequently discussed as plausible contributors to cumulative tissue loading and technique changes that may increase vulnerability [5,8].

Despite the practical importance of throwing under fatigue, the evidence base in handball remains fragmented. Systematic reviews have addressed throwing speed broadly—summarizing determinants, kinematic correlates, and training-related improvements—but they do not specifically synthesize experimentally induced fatigue effects on throwing velocity and accuracy, nor do they provide pooled effect estimates for fatigue-related changes [10,11,12]. Meanwhile, primary studies in handball have used heterogeneous fatigue models, including simulated game activity protocols, localized upper-limb fatigue protocols, and physiological fatigue manipulations, reporting variable impacts on ball velocity and/or accuracy [13,14,15]. Such variability is expected because the magnitude and direction of fatigue effects can depend on the fatigue stimulus (local vs. whole-body), participant characteristics (age, sex, or competitive level), and outcome definitions (radar-measured velocity, target-based accuracy metrics, or composite indices) [5,7]. In addition, many laboratory studies in this domain are small, which increases uncertainty and makes quantitative synthesis particularly valuable. Previous handball research has consistently identified throwing velocity as a key performance indicator, with ball speed influenced by throwing technique, playing level, strength–power capacity, and the type of throwing task [3,10]. Prior reviews have also shown that training interventions, particularly resistance- and power-based programs, can improve throwing velocity in handball players [11,12]. However, these reviews have primarily focused on determinants or training-induced improvements in throwing performance rather than on acute fatigue-related decrements [10,11,12]. Individual experimental studies have reported variable fatigue effects, ranging from minimal changes in throwing velocity after some whole-body fatigue protocols to marked reductions after more intensive handball-specific or localized upper-limb fatigue protocols [13,14,15]. Similarly, accuracy findings have been inconsistent, partly because studies have used different accuracy-related metrics and scoring approaches [15].

Therefore, a systematic review and meta-analysis focusing specifically on handball players is warranted to clarify whether fatigue meaningfully affects throwing velocity and/or accuracy, and to estimate the magnitude of these effects across study designs and fatigue protocols. The present review is reported in accordance with PRISMA 2020 guidance, which emphasizes transparent reporting of the rationale, objectives, and methods underpinning evidence synthesis [16]. The objective of this systematic review and meta-analysis is to determine the effect of fatigue on throwing performance in handball players, specifically examining outcomes such as throwing/ball/release velocity and throwing/shooting accuracy. Where data allow, we also aim to explore whether observed effects differ according to the type of fatigue protocol (e.g., localized upper-limb fatigue, whole-body protocols, or simulated match activity) and participant profile (e.g., age group or competitive level) [7,16].

## 2. Materials and Methods

### 2.1. Study Design

This study was designed as a systematic review and meta-analysis investigating the effect of fatigue (e.g., localized upper-limb fatigue, whole-body fatigue protocols, or simulated match activity) on throwing performance outcomes (e.g., throwing/ball/release velocity and throwing/shooting accuracy) in handball players. The review was conducted and reported in accordance with the Preferred Reporting Items for Systematic Reviews and Meta-Analyses (PRISMA) 2020 statement. The review protocol was prospectively registered in the International Prospective Register of Systematic Reviews (PROSPERO) (Registration number: CRD420251161660) to enhance transparency and minimize risk of selective reporting and unnecessary duplication [17,18].

### 2.2. Eligibility Criteria

Eligibility criteria were pre-specified using a PECOS framework (Participants, Exposure, Comparator, Outcomes, Study design) [16,19]. Eligible studies included human team handball players of any sex, age, and competitive level; mixed-sport studies were included only when handball-specific data were reported separately or were extractable [19]. For mixed-sport samples, only the handball-specific subgroup data were extracted; studies were excluded when outcomes could not be separated for handball players. Studies were required to examine an acute fatigue condition (e.g., localized upper-limb fatigue, whole-body protocols, or simulated match activity) and include an appropriate non-fatigued comparator, such as pre–post fatigue assessments or fatigued versus non-fatigued conditions. Inclusion further required at least one quantitative throwing-performance outcome relevant to handball, specifically throwing/ball/release velocity and/or throwing or shooting accuracy, assessed via any valid handball throwing task. Experimental or quasi-experimental designs capable of isolating fatigue effects were eligible. Studies were excluded if they did not involve handball players (or extractable handball data), lacked an acute fatigue exposure, or did not report eligible throwing outcomes. Only peer-reviewed English-language journal articles were included, with no restriction on publication year.

### 2.3. Information Sources and Search Strategy

A comprehensive literature search was performed to identify studies examining the effect of fatigue on throwing performance in handball players. The primary electronic databases searched were PubMed (NCBI), MEDLINE Complete (EBSCO), CINAHL (EBSCO), and Scopus. SPORTDiscus was also considered because of its relevance to sports science; however, it was not included because institutional access was not available to the authors at the time of the search. The search was conducted from database inception to the final search date (24 January 2026), without restrictions on publication year, and was limited to studies published in English. Search strategies were tailored to each database by combining free-text terms and, where applicable, controlled vocabulary (e.g., MeSH terms in PubMed and subject headings in EBSCO) in line with recommended systematic-review methods [14,19].

The search strategy used Boolean operators to combine three core concepts: fatigue, throwing performance, and handball. Specifically, fatigue-related terms included “fatigue”, “muscle fatigue”, “neuromuscular fatigue”, “exertion”, and “exercise-induced fatigue”. Throwing-performance terms included “throwing velocity”, “ball velocity”, “ball speed”, “release velocity”, “throwing accuracy”, “shot accuracy”, “accuracy”, “precision”, and related variants. The sport-specific concept included “handball” and “team handball”. An example of the core structure applied across databases was (fatigue OR muscle fatigue OR neuromuscular fatigue OR exertion OR exercise-induced fatigue) AND (throwing velocity OR ball velocity OR ball speed OR release velocity OR throwing accuracy OR shot accuracy OR accuracy OR precision) AND (handball OR team handball). Database-specific syntax, truncation, and field tags were applied as needed to maximize sensitivity while maintaining relevance.

To reduce the risk of missing eligible studies, supplementary searches were also performed by screening the reference lists of included papers and relevant reviews, as well as by conducting forward citation tracking. In addition, Google Scholar was used as a complementary source to identify potentially relevant records not indexed in the main databases, consistent with guidance to consider additional sources beyond bibliographic databases when appropriate. The supplementary search strategy, including Google Scholar screening and backward and forward citation tracking, was used to minimize the possibility of missing relevant sports science studies that might have been indexed in databases not directly accessible to the authors. All records were exported to a reference-management workflow, and duplicates were removed prior to screening.

### 2.4. Study Selection Procedure

All records retrieved from the database searches were exported and collated, and duplicates were removed prior to screening. Study selection was conducted in two stages in accordance with PRISMA 2020 guidance [14]. First, titles and abstracts were screened against the predefined eligibility criteria to exclude clearly irrelevant records. Second, the full texts of potentially eligible articles were retrieved and assessed for inclusion. Reasons for full-text exclusions were documented.

The study selection process was performed independently by two reviewers. Disagreements at any stage were resolved through discussion, and when consensus could not be reached, a third reviewer arbitrated. The overall selection process and numbers of records at each stage were documented and presented using a PRISMA flow diagram.

### 2.5. Data Extraction

Data extraction was performed using a standardized, pilot-tested extraction form developed a priori to ensure consistency across studies. For each included study, the following information was extracted: bibliographic details (authors, year), participant characteristics (sample size, sex, age, playing level), study design and setting, details of the fatigue protocol (type, duration, intensity and verification when reported), characteristics of the throwing task (e.g., standing/jump shot, number of trials, target specification), and outcome measures related to throwing performance (throwing/ball/release velocity and/or throwing/shooting accuracy). When studies reported multiple fatigue conditions, time points, or throwing tasks, data were extracted for all eligible comparisons and pre-specified outcomes. Velocity-related outcomes were reported using terms such as ball speed, ball velocity, or release velocity across studies; for consistency, these were collapsed as throwing velocity in the synthesis. Accuracy-related outcomes were reported as hit counts/percentages, points-based scores, or spatial error (e.g., cm deviation); these were collectively referred to as throwing accuracy, while retaining each study’s original metric definition in the tables.

For quantitative synthesis, means and measures of dispersion (standard deviations, standard errors, or confidence intervals) were extracted for fatigued and non-fatigued conditions (or pre- and post-fatigue). When numerical data were not directly reported in tables or text, values were derived from figures using digital extraction methods when feasible, or authors were contacted to request missing data, following established guidance for systematic reviews. Any uncertainties in extraction were resolved by re-checking the original reports and, when necessary, through discussion among the review team to ensure accuracy and completeness.

### 2.6. Risk of Bias Assessment

Risk of bias was assessed at the outcome level using established methodological tools appropriate to the design of each included study. For randomized studies (including crossover randomized designs where applicable), the Cochrane Risk of Bias tool version 2 (RoB 2) was used, applying the relevant RoB 2 variant for crossover trials when needed [18,20,21]. For non-randomized studies of interventions (e.g., quasi-experimental pre–post designs without random allocation), the Risk Of Bias In Non-randomized Studies—of Interventions tool (ROBINS-I) was applied [22]. These tools evaluate bias across key domains, such as the randomization process or confounding, deviations from intended conditions/interventions, missing outcome data, outcome measurement, and selective reporting, leading to an overall risk-of-bias judgement for each effect estimate [20,22].

Two reviewers (SH and ICT) independently completed the risk-of-bias assessments. Any disagreements were resolved by discussion, and where consensus could not be reached, a third reviewer (MAE) adjudicated. Risk-of-bias judgements were incorporated into the interpretation of findings and, where meta-analysis was conducted, were used to inform sensitivity analyses that examined the robustness of pooled estimates to exclusion of studies judged to be at high (RoB 2) or serious/critical (ROBINS-I) risk of bias.

### 2.7. Data Synthesis

A qualitative synthesis was first undertaken to summarize study characteristics and findings, with particular attention to the type of fatigue protocol (localized upper-limb vs. whole-body vs. simulated match activity), the throwing task (e.g., standing, running, or jump shot), and outcome definitions (velocity-related outcomes and accuracy-related outcomes). Where at least two studies reported sufficiently comparable outcomes and provided extractable quantitative data, meta-analyses were conducted in accordance with methodological guidance for systematic reviews [19,23].

Because throwing outcomes were reported using different units, protocols, and accuracy metrics across studies, pooled effects were calculated using the standardized mean difference corrected for small-sample bias (Hedges’ g) with 95% confidence intervals. Effect sizes were oriented so that negative values indicated a reduction in performance under fatigue (i.e., lower velocity and/or poorer accuracy) relative to the non-fatigued condition. For accuracy metrics expressed as error or deviation (where lower values indicate better performance), values were multiplied by −1 prior to effect-size calculation so that, across all accuracy measures, higher values consistently reflected better accuracy. This ensured that negative Hedges’ g values uniformly represented poorer performance under fatigue for both velocity and accuracy outcomes. Velocity and accuracy outcomes were synthesized in separate meta-analyses to avoid conflating constructs.

For repeated-measures (pre–post) designs, effect sizes were computed from standardized pre–post change scores using paired-data approaches that account for within-participant dependence. When change-score standard deviations or pre–post correlations were not reported, the pre–post correlation required for paired variance estimation was imputed using a conservative, prespecified value of r = 0.70 and explored via sensitivity analyses across plausible alternative correlation values [19,24]. The randomized crossover study was similarly treated as a within-participant comparison, using the RoB 2 tool for crossover trials and paired-data effect estimation where appropriate.

When a study reported multiple eligible throwing conditions or time points, a single comparison was selected per construct for the primary analysis to minimize unit-of-analysis errors, prioritizing (i) the most sport-specific throwing task and (ii) the time point most representative of the post-fatigue state. When multiple equivalent measures were reported for the same construct, priority was given to measures most consistently reported across studies.

Given expected clinical and methodological heterogeneity across fatigue protocols and participant populations, pooled estimates were calculated using a random-effects model. Heterogeneity was interpreted in relation to prespecified clinical and methodological sources, including the type of fatigue protocol, throwing task, participant characteristics, and outcome definition. In particular, localized upper-limb fatigue protocols were considered separately from whole-body or simulated match-activity protocols because they may affect throwing performance through different physiological and biomechanical pathways. For accuracy/precision outcomes, heterogeneity was expected to be especially high because studies used different operational definitions, including hit counts, success rates, points-based scores, and spatial error. Therefore, pooled accuracy estimates were interpreted cautiously and were considered exploratory when outcome definitions were not sufficiently comparable. Between-study heterogeneity was quantified using τ^2^ and I^2^ and assessed using Cochran’s Q [17,21]. In addition to pooled effect sizes and 95% confidence intervals, 95% prediction intervals were reported, where appropriate, to reflect the expected range of true effects in comparable future studies. Meta-analyses were conducted in R software (R Foundation for Statistical Computing, Vienna, Austria) using the metafor package for statistical analyses [25]. Data import, processing, and export were performed using the readxl, dplyr, and writexl packages, respectively. 

### 2.8. Subgroup and Sensitivity Analyses

Planned subgroup analyses were conducted when sufficient data were available to explore whether the effect of fatigue on throwing performance differed according to prespecified study and participant characteristics. Subgroups were defined a priori based on fatigue stimulus and ecological relevance, separating localized upper-limb/shoulder fatigue protocols from whole-body or handball-specific simulated match activity protocols, because these models can differ in physiological mechanisms and their expected impact on throwing mechanics. Where data permitted, additional subgroup analyses were conducted by age group (adolescent vs. adult) and by throwing task (jump shot vs. other), reflecting potential differences in technique, strength capacity, and match demands across populations and task constraints.

Sensitivity analyses were used to evaluate the robustness of pooled estimates to methodological decisions and assumptions. First, analyses were repeated after excluding studies judged to be at high risk (RoB 2) or serious/critical risk (ROBINS-I) of bias to assess the influence of study quality on summary effects. Second, because several studies did not report the pre–post correlation required for paired effect-size variance estimation, sensitivity analyses were conducted using alternative plausible correlation values to test whether conclusions were dependent on the imputed correlation. Third, where a study reported multiple eligible measures for the same construct (e.g., multiple accuracy indices or throwing conditions), alternative selection rules were tested to confirm that the primary conclusions were not driven by outcome choice or unit-of-analysis decisions. Fourth, because one study [26] reported outcomes at the throw (shot) level rather than as participant-level summaries (raising potential unit-of-analysis concerns), meta-analyses were repeated excluding this study to evaluate the impact of possible clustering of repeated throws within players on pooled estimates.

### 2.9. Certainty of Evidence and Publication Bias

The certainty of evidence for each primary outcome (throwing/ball/release velocity and throwing/shooting accuracy) was evaluated using the GRADE approach [27,28,29]. Certainty ratings were made at the outcome level and summarized as high, moderate, low, or very low. Evidence was downgraded based on risk of bias, inconsistency, indirectness, imprecision, and publication bias, following GRADE guidance. Where meta-analysis was performed, the GRADE assessment incorporated the magnitude and precision of pooled effects, heterogeneity, and the methodological quality of contributing studies. When at least 10 studies contributed to a given meta-analysis, small-study effects were explored using funnel plots. Formal tests for funnel plot asymmetry were conducted as described below [19,30]. When asymmetry suggested possible reporting bias, sensitivity analyses examined robustness, and, where feasible, the trim-and-fill method was used as an exploratory approach to estimate the potential influence of missing studies on pooled effects [19,31].

## 3. Results

### 3.1. Study Selection Results

**Figure 1:**
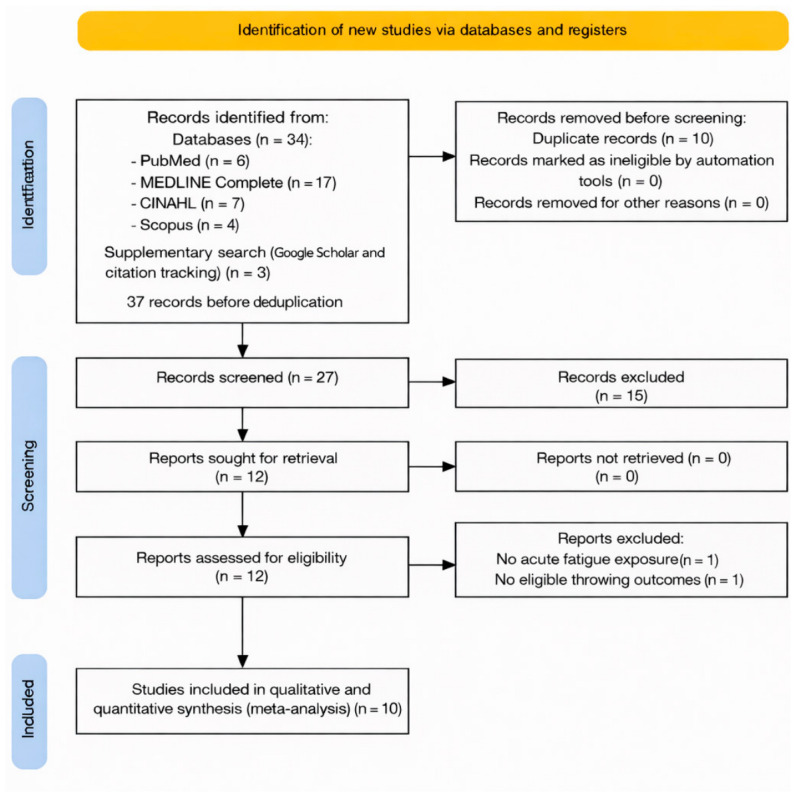
PRISMA 2020 flow diagram of study selection.

The database search identified 34 records in total (PubMed, n = 6; MEDLINE Complete, n = 17; CINAHL, n = 7; Scopus, n = 4). An additional three records were identified through supplementary searching (Google Scholar and citation tracking), yielding 37 records before deduplication. After removing 10 duplicates, 27 unique records remained for title and abstract screening. Of these, 15 records were excluded as clearly irrelevant to the review question. The full texts of 12 articles were assessed for eligibility. Two full-text articles were excluded (n = 2): one because it did not include an acute fatigue exposure (i.e., no experimentally induced fatigue condition), and one because it did not report eligible throwing-performance outcomes (throwing/ball/release velocity and/or throwing/shooting accuracy), despite involving handball players. Consequently, 10 studies were included in the qualitative synthesis. For quantitative synthesis, 10 comparisons contributed to the throwing-velocity meta-analysis and 6 comparisons contributed to the throwing-accuracy meta-analysis. The selection process is presented in the PRISMA 2020 flow diagram (Figure 1).

### 3.2. Characteristics of Included Studies

The 10 included studies [13,14,15,26,32,33,34,35,36,37], published between 2007 and 2025, examined the acute effects of fatigue on throwing-related performance in handball players. The outcomes of interest focused primarily on throwing, ball, or release velocity and on throwing or shooting accuracy, although not all studies reported both outcomes [15,26,32,33]. Most studies employed within-participant pre–post designs, in which throwing performance was assessed immediately before and after a fatiguing stimulus, whereas one study used a randomized crossover design to compare two different work–rest distributions during simulated match play [34]. Across studies, throwing velocity was typically quantified as radar-derived ball speed or release velocity, whereas accuracy was assessed using target-based outcomes, including successful hits, hit percentages, and spatial error relative to a predefined target area [15,26,32,35].

Participants ranged from adolescent to adult competitive handball players and included both male-only and female-only samples, with further variation in playing level and competitive background across studies [13,14,15,26,32,33]. Sample sizes were generally modest, ranging from 8 players in the simulated match-play crossover study [34] to 30 elite female players in a repeated-throwing fatigue study that also accounted for shoulder pain status [33]. Several studies focused on adolescent players [14,26], whereas others examined adult competitive or elite players performing standardized throwing tasks under either controlled laboratory-style fatigue protocols or more sport-specific conditions [13,15,32,33,35,36,37].

The fatigue-induction methods were heterogeneous and broadly grouped into ecologically oriented and controlled protocols. Ecologically oriented approaches sought to reproduce handball-specific or match-related demands through simulated game activities, structured match-play simulations, or progressive handball circuits, with throwing performance assessed either immediately after the workload or repeatedly across stages to capture the progressive effects of fatigue [13,15,32,34]. By contrast, controlled protocols induced fatigue through standardized or localized loading designed to stress the upper body or the whole body in a reproducible manner, including metronome-paced push-up fatigue [14], treadmill running to exhaustion [36], localized medicine-ball throwing to volitional fatigue [37], and the 30–15 intermittent fitness test used as a field-based intermittent running protocol [26,35]. One additional study applied a functional repeated-throwing protocol consisting of submaximal and maximal standing throws, thereby examining fatigue under a sport-specific exposure while also accounting for shoulder pain status [33].

The throwing tasks also varied across studies and included standing throws, jump shots, overarm target-shooting tests, and repeated shooting protocols performed under progressively fatiguing conditions [13,14,15,26,32,33,34,35,36,37]. The timing of assessment likewise differed, with some studies using a single immediate post-fatigue test and others employing repeated measurements across successive time blocks or workload stages [15,32,34]. This methodological diversity is important, as it may influence both the magnitude and the direction of the observed fatigue effects, particularly for accuracy-related outcomes, which were operationalized less consistently than throwing-velocity outcomes [15,26,32,35].

Reporting of throwing-task reliability was inconsistent across the included studies. Throwing velocity was commonly assessed using radar-based or instrumented methods, which are generally considered objective approaches; however, few studies reported protocol-specific reliability indices, such as intraclass correlation coefficients, coefficients of variation, standard error of measurement, or minimal detectable change. Reliability information was even less consistently reported for accuracy outcomes, which were assessed using heterogeneous metrics, including hit counts, percentage accuracy, points-based scores, and spatial error. This limits confidence in distinguishing true fatigue-related changes from measurement variability, particularly for accuracy outcomes.

Overall, the included literature demonstrated substantial heterogeneity in fatigue operationalization, throwing-task selection, outcome definition, and assessment timing. In addition, certain study-specific features were relevant to the interpretation of the evidence, such as throw-level rather than participant-level outcome summaries in one study [26] and subgroup-based reporting according to pain status in another [33]. This heterogeneity was taken into account when structuring the quantitative synthesis, interpreting pooled estimates and the certainty of evidence, and conducting subgroup and sensitivity analyses.

A summary of study design, participant characteristics, key outcomes, and effect sizes is presented in Table 1, while the fatigue-induction procedures used across studies are summarized in Table 2.

### 3.3. Risk of Bias Within Studies

Risk-of-bias assessments are summarized in Table 3. Section A presents the domain-level ROBINS-I judgments for the nine non-randomized studies, whereas Section B presents the RoB 2 assessment for the randomized crossover study. The single randomized crossover study [34] was assessed using the RoB 2 crossover variant and was judged as presenting some concerns, mainly because potential carryover effects could not be ruled out given the limited reporting on washout adequacy and period effects. The remaining nine studies were non-randomized pre–post or repeated-measures designs and were assessed using the ROBINS-I tool.

Across the non-randomized studies, the most important source of potential bias was confounding, reflecting the absence of random allocation and parallel control groups, which left results vulnerable to time-related effects, pacing or learning effects, and uncontrolled variation in prior training load, recovery status, or participant characteristics. In most studies, bias related to selection of participants and missing outcome data was judged as low. Bias arising from deviations from intended interventions was generally low to moderate, although standardization of pre-test conditions and procedural control were not always fully reported. Bias in measurement of outcomes was mostly low, likely because many studies used objective instrumentation such as radar devices, motion-capture systems, inertial sensors, or electromyography; however, assessor blinding was rarely described and may be more relevant for accuracy-related outcomes than for velocity-based measures. Bias in selection of the reported result was frequently judged as moderate because study protocols or preregistration records were generally unavailable.

At the domain level, judgments were predominantly low to moderate, although serious concerns were identified in selected studies, particularly in relation to confounding and, in one case, selection of the reported result. One study involving subgroup-based comparisons according to pain status showed serious concern for confounding [33], and another study also showed serious concerns in specific ROBINS-I domains [15]. No study was judged to be at critical risk of bias.

These judgements were taken into account when interpreting the evidence and, where a meta-analysis was performed, sensitivity analyses excluded studies at high risk of bias in RoB 2 or at serious/critical risk of bias in ROBINS-I.

### 3.4. Meta-Analysis: Throwing Velocity

**Figure 2:**
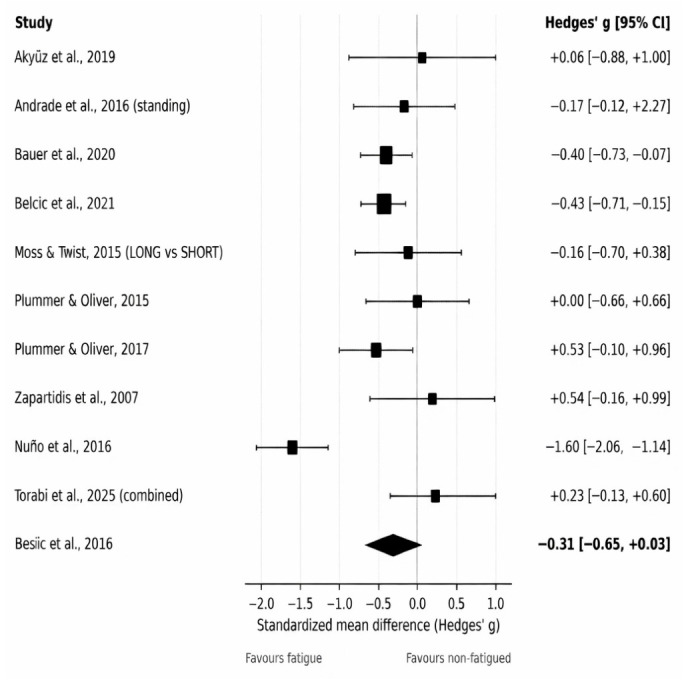
Forest plot of the effect of fatigue on throwing velocity. Random-effects meta-analysis showing standardized mean differences (Hedges’ g) and 95% confidence intervals for the effect of fatigue on throwing/ball/release velocity. Negative values indicate reduced throwing velocity under fatigued conditions. The pooled effect and between-study heterogeneity (I^2^, τ^2^) are shown. Studies included in the analysis: [13,14,15,26,32,33,34,35,36,37].

The primary meta-analysis of throwing/ball/release velocity included 10 comparisons (k = 10). Where subgroup-specific estimates were available within the same study, a single combined comparison was used to avoid double counting. Under a random-effects model (DerSimonian–Laird), fatigue was associated with a small-to-moderate reduction in throwing velocity, although the confidence interval included the null (Hedges’ g = −0.31, 95% CI −0.65 to 0.03; Figure 2). Substantial heterogeneity was present (I^2^ = 77.8%, τ^2^ = 0.219, Q(9) = 40.59, *p* < 0.001), consistent with variation in fatigue protocols (localized vs. whole-body vs. match-simulated), throwing tasks, and populations. The 95% prediction interval ranged from −1.30 to 0.67, suggesting substantial between-study variability and indicating that the true effect in a comparable future study could range from a meaningful reduction in throwing velocity to little or no impairment.

Although the overall direction of effect generally indicated reduced velocity under fatigue, study-specific effects varied markedly, with one comparison showing a very large negative effect and several showing small or near-zero effects.

### 3.5. Meta-Analysis: Throwing Accuracy

**Figure 3:**
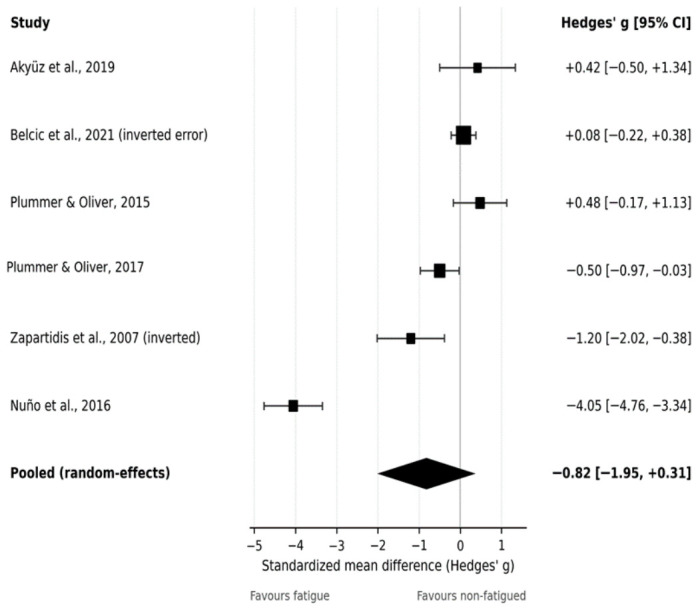
Forest plot of the effect of fatigue on throwing accuracy. Random-effects meta-analysis showing standardized mean differences (Hedges’ g) and 95% confidence intervals for the effect of fatigue on throwing accuracy. Accuracy outcomes were oriented so that negative values represent poorer performance under fatigue. The pooled estimate and heterogeneity statistics are presented. Studies included in the analysis: [15,26,32,35,36,37].

The primary meta-analysis of throwing accuracy included six comparisons (k = 6), with accuracy metrics oriented so that negative values indicated poorer accuracy under fatigue. The random-effects pooled estimate suggested a negative effect of fatigue on accuracy, although uncertainty was substantial because of extreme heterogeneity (Hedges’ g = −0.82, 95% CI −1.95 to 0.31; Figure 3). Because accuracy/precision was operationalized differently across studies, the pooled estimate should not be interpreted as a single definitive effect. Although all outcomes were statistically oriented in the same direction before synthesis, the underlying constructs were not fully equivalent. For example, hit counts and success percentages reflect outcome success, whereas spatial error and points-based scoring may capture different aspects of precision, target proximity, or task constraint. Therefore, the accuracy meta-analysis should be interpreted as an exploratory synthesis indicating possible context-specific impairment rather than as evidence of a uniform fatigue effect on accuracy. Heterogeneity was very high (I^2^ = 95.8%, τ^2^ = 1.886, Q(5) = 118.94, *p* < 0.001), likely reflecting major differences in accuracy definitions (e.g., hits, error distance, points) and the presence of at least one extremely large effect estimate. The 95% prediction interval ranged from −3.79 to 2.21, indicating substantial uncertainty regarding the likely effect in comparable future studies and suggesting that the true effect could range from a large reduction in accuracy to little effect or even improvement.

Overall, the evidence suggests that fatigue may impair throwing accuracy in some contexts, but pooled inference is limited by heterogeneity and the influence of extreme effect estimates.

### 3.6. Subgroup Analyses

Prespecified subgroup analyses were conducted to explore potential sources of heterogeneity in the velocity meta-analysis (Figure 2). When stratified by fatigue type, localized upper-limb/shoulder fatigue protocols (k = 2) showed a statistically significant reduction in throwing velocity under fatigue (fixed-effect model due to very small k: g = −0.44, 95% CI −0.71 to −0.17). In contrast, studies using whole-body or match-simulated/functional fatigue protocols (k = 8) showed a non-significant pooled effect with substantial heterogeneity (random-effects: g = −0.25, 95% CI −0.72 to 0.22; I^2^ = 82.6%, τ^2^ = 0.359).

Subgroup analysis by age group indicated larger and statistically significant fatigue-related decrements among adolescent players (k = 2; fixed-effect: g = −0.35, 95% CI −0.64 to −0.05), whereas the pooled effect in adult samples (k = 8) was not statistically significant and remained highly heterogeneous (random-effects: g = −0.31, 95% CI −0.76 to 0.14; I^2^ = 82.5%, τ^2^ = 0.323).

Finally, subgroup analyses by throwing task suggested that jump-shot performance may be more consistently affected by fatigue than other throwing tasks. Specifically, jump-shot outcomes (k = 4) showed a statistically significant reduction in velocity with minimal heterogeneity (random-effects: g = −0.37, 95% CI −0.59 to −0.16; I^2^ = 0%, τ^2^ = 0), whereas other throwing tasks (k = 6) showed a non-significant pooled effect with marked heterogeneity (random-effects: g = −0.30, 95% CI −0.92 to 0.31; I^2^ = 86.9%, τ^2^ = 0.490). Additional subgroup analyses for accuracy/precision were not considered sufficiently meaningful because only six comparisons contributed to this outcome and the included studies differed substantially in how accuracy was defined and scored. Stratifying these already limited data by fatigue type, task, or participant characteristics would have produced very small subgroups and potentially misleading estimates. Therefore, accuracy findings were interpreted narratively with emphasis on outcome-definition heterogeneity rather than on formal subgroup pooling (Figure 3).

### 3.7. Sensitivity Analyses

Sensitivity analyses were conducted to evaluate the robustness of the pooled estimates to influential comparisons and analytic assumptions for velocity and accuracy (Figure 2 and Figure 3). For throwing velocity, excluding the study that contributed the combined pain/no-pain comparison yielded a clearer negative pooled effect (random-effects: g = −0.40, 95% CI −0.74 to −0.06; I^2^ = 74.3%, τ^2^ = 0.183; Q(8) = 31.11, *p* < 0.001; k = 9). In addition, excluding the study with the largest negative velocity effect reduced between-study heterogeneity and produced a smaller but still statistically significant pooled effect (random-effects: g = −0.21, 95% CI −0.42 to −0.01; I^2^ = 29.4%, τ^2^ = 0.0267; Q(8) = 11.34, *p* = 0.18; k = 9). Overall, these analyses indicated that the direction of effect for velocity was generally stable, with fatigue tending to reduce velocity, although the magnitude and statistical significance were partially influenced by specific comparisons (Figure 2).

For accuracy, given the presence of an extreme effect estimate in one study, a sensitivity analysis excluding this comparison substantially attenuated the pooled effect and reduced heterogeneity, while the confidence interval remained wide (random-effects: g = −0.18, 95% CI −0.74 to 0.38; I^2^ = 70.8%; k = 5), indicating that pooled inference was sensitive to this outlying study (Figure 3). Collectively, these sensitivity analyses suggest that conclusions for velocity are more robust than those for accuracy.

Because Belčić et al. [26] reported outcomes at the throw level rather than as participant-level summaries, we repeated the meta-analysis excluding this study to assess the potential influence of unit-of-analysis issues. Excluding this study did not materially change the direction or magnitude of the pooled effects, and the overall conclusions remained unchanged.

### 3.8. Publication Bias and Small-Study Effects

**Figure 4:**
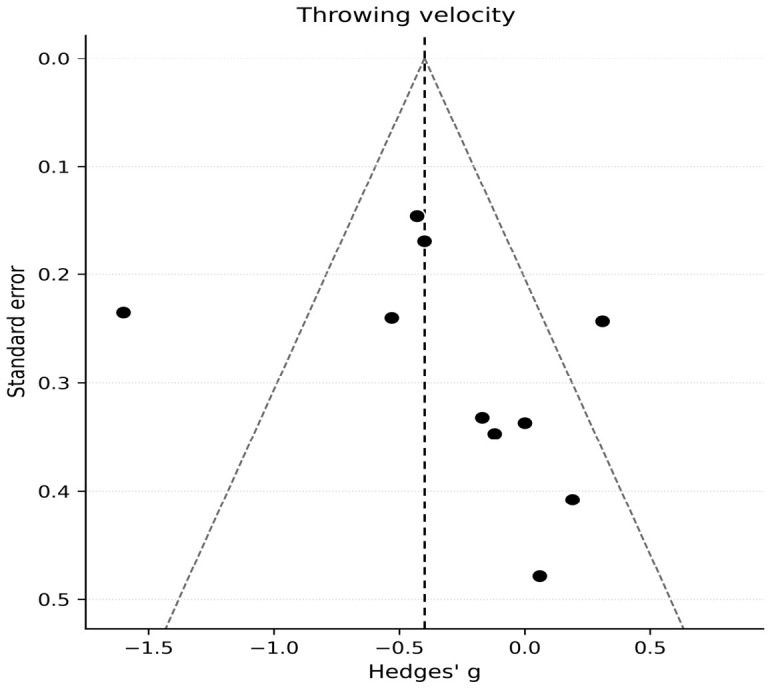
Funnel plot for assessment of small-study effects and publication bias in throwing velocity outcomes. Effect sizes (Hedges’ g) are plotted against their standard errors. Visual inspection and Egger’s regression test were used to evaluate potential asymmetry and small-study effects. The dots represent the individual study effect sizes, and the dashed line indicates the pooled effect estimate. Studies included in the analysis: [13,14,15,26,32,33,34,35,36,37].

For velocity (k = 10), visual inspection of the funnel plot did not suggest marked asymmetry, although interpretation remains cautious given the modest number of comparisons (Figure 4). Egger’s regression test did not suggest statistically significant small-study effects (*p* = 0.398).

**Figure 5:**
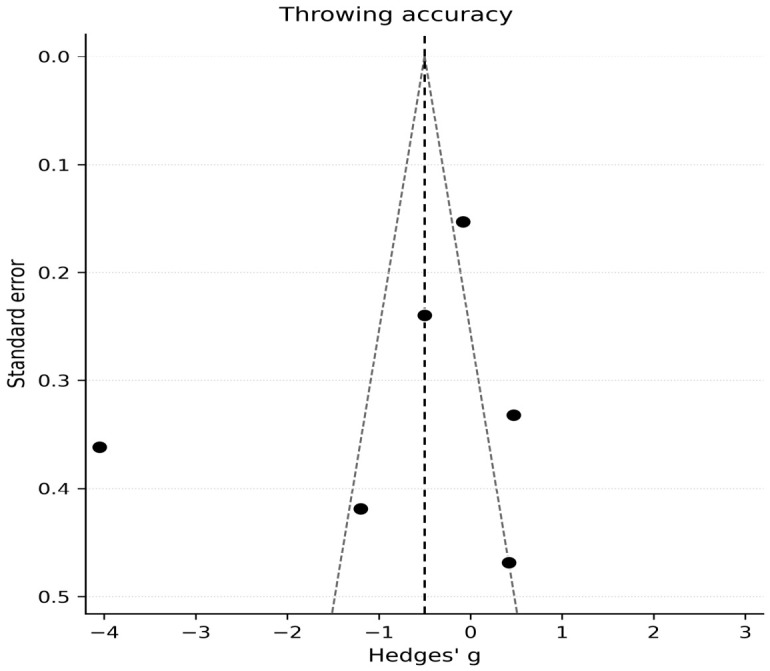
Funnel plot for assessment of small-study effects and publication bias in throwing accuracy outcomes. Effect sizes (Hedges’ g) are plotted against their standard errors. Funnel plot interpretation was considered exploratory due to the limited number of contributing studies. The dots represent the individual study effect sizes, and the dashed line indicates the pooled effect estimate. Studies included in the analysis: [15,26,32,35,36,37].

For accuracy (k = 6), methods for assessing publication bias or small-study effects were considered of limited interpretability because fewer than 10 comparisons contributed to the meta-analysis. Accordingly, funnel plot inspection was treated as exploratory, and formal asymmetry testing was not emphasized (Figure 5).

Trim-and-fill was not applied/interpreted because of the limited number of comparisons and the exploratory nature of these analyses.

### 3.9. Certainty of Evidence (GRADE)

For throwing velocity, certainty of evidence was rated as moderate. This rating was downgraded mainly for inconsistency, due to substantial heterogeneity across fatigue protocols and throwing tasks, and for study limitations/risk of bias, because most contributing evidence came from non-randomized repeated-measures designs. Although the overall direction of effect generally indicated reduced velocity under fatigue, precision was limited in some comparisons and confidence intervals varied across studies.

For throwing accuracy, certainty of evidence was rated as low. This reflected very serious inconsistency, including extreme heterogeneity and substantial variation in accuracy definitions, together with imprecision arising from wide confidence intervals around the pooled estimate and additional study limitations. The diversity of outcome metrics and the influence of outlying effects further reduced confidence in the pooled estimate.

## 4. Discussion

This systematic review and meta-analysis examined whether acute fatigue impairs handball throwing performance, focusing on throwing velocity and accuracy. The quantitative synthesis suggested that fatigue is associated with a small-to-moderate reduction in throwing velocity, albeit with substantial heterogeneity across studies and protocols. For accuracy, the pooled estimate suggested a possible deterioration under fatigue, but inference was much less stable due to extreme heterogeneity in how “accuracy” was defined and measured and because the pooled effect was sensitive to outlying results. This finding should be interpreted with particular caution. The very high heterogeneity for accuracy/precision suggests that the pooled estimate may not represent a single common construct across studies. Accuracy was measured using different indicators, including hit counts, success percentages, points-based scoring systems, and spatial error, and these outcomes may reflect different aspects of shooting performance. Consequently, the accuracy synthesis should be viewed as exploratory and hypothesis-generating rather than as a definitive pooled estimate. Taken together, the evidence supports the interpretation that fatigue may reduce throwing performance, but the magnitude and even detectability of the effect depend strongly on the fatigue stimulus, the throwing task, and the operational definition of performance.

A key implication is that the overall pooled effects should be interpreted as “average tendencies across heterogeneous conditions,” rather than as a single universal estimate of how fatigue affects throwing. This aligns with the contemporary understanding of fatigue as a task-dependent phenomenon with multiple mechanisms and site-specific contributions (central vs. peripheral), where the performance consequences depend on the interaction between the fatigued systems and the motor task demands [5]. In handball, throwing is a high-velocity, coordination-intensive skill requiring rapid force production and effective proximal-to-distal sequencing; therefore, the same fatigue exposure may affect velocity and accuracy differently, and athletes may partially compensate in one dimension at the expense of the other [38]. The wide prediction intervals further support this interpretation, suggesting that the effect of fatigue may vary meaningfully across protocols, player samples, and task characteristics, which is consistent with the substantial between-study heterogeneity observed in both meta-analyses.

### 4.1. Interpretation of Throwing Velocity Findings

The pooled reduction in velocity is practically plausible and may be meaningful in competitive handball because higher ball speed constrains goalkeeper response time and is a core determinant of successful attacking outcomes [10]. Although the meta-analytic estimate is expressed as a standardized mean difference, its practical relevance is supported indirectly by training-focused syntheses showing that targeted conditioning—particularly resistance training—can meaningfully increase throwing velocity [11,39]. Against that background, even a small-to-moderate standardized decrement under fatigue could be performance-relevant, especially during decisive match periods when fatigue accumulates and shot quality becomes more variable.

The heterogeneity observed in the velocity meta-analysis is not surprising given the diversity of fatigue models. Localized upper-limb/shoulder fatigue protocols and ecologically valid match-simulation protocols likely stress different limiting factors. Localized protocols may preferentially reduce the capacity of key upper-limb and trunk musculature involved in ball acceleration and stabilization, thereby lowering peak velocity more consistently, while whole-body or simulated match fatigue may alter pacing, neuromuscular coordination, and decision-making constraints in ways that vary by athlete and context. This task-specific mechanism view is consistent with the foundational fatigue literature: there is no single mechanism responsible for fatigue, and the causes and consequences of fatigue differ across tasks and conditions [5]. The more consistent velocity impairment observed in jump-shot conditions is also coherent: jump shots place higher demands on whole-body coordination, timing, and trunk–shoulder coupling and therefore may be more sensitive to fatigue-related disruptions than simpler throwing conditions. These findings support a “specificity” interpretation: fatigue effects may be more readily detected when the task is sport-specific and coordination-intensive. Based on the subgroup and sensitivity analyses, throwing velocity appeared more consistently reduced after localized upper-limb fatigue protocols and during jump-shot tasks. This suggests that velocity may be particularly vulnerable when fatigue directly affects the throwing arm/shoulder complex or when the task requires high levels of whole-body coordination.

### 4.2. Interpretation of Throwing Accuracy Findings

Accuracy findings require more cautious interpretation. Across studies, accuracy was assessed using different constructs (hit rate, spatial error/deviation, or points-based scores), which are only partially interchangeable. Even after orienting outcomes to a common direction, this measurement diversity likely contributed substantially to the very high heterogeneity and the sensitivity of the pooled estimate to extreme effects. Therefore, the most defensible interpretation is not that fatigue produces a uniform reduction in accuracy across contexts, but rather that fatigue may impair accuracy in certain settings and under certain definitions of accuracy, while in other contexts, accuracy may be preserved—potentially through compensatory strategies such as adjusting release parameters, prioritizing placement over speed, or increasing attentional investment. The wider team-sport literature supports this nuanced picture: systematic reviews in soccer report that physical fatigue tends to impair technical performance overall, but effects vary by the specific technical skill and protocol characteristics [40]. Similarly, meta-analytic work on mental fatigue in soccer indicates measurable decrements in technical skills, again with outcome- and task-dependence [41]. While these reviews are not handball throwing–specific and involve different fatigue constructs, they reinforce the broader principle that technical outcomes are heterogeneous and sensitive to how performance is operationalized. In contrast, accuracy/precision appeared most affected in studies using constrained target-based or spatial-error outcomes under intensive handball-specific fatigue conditions. However, this interpretation remains tentative because of the small number of studies and substantial inconsistency in measurement approaches.

### 4.3. How These Findings Fit Within Handball Performance Research

Handball-specific reviews emphasize that throwing speed is a key performance variable and a central target of training interventions [10,11,39]. The present review complements that literature by focusing not on how to increase velocity through training, but on how acute fatigue may erode velocity and/or accuracy in the short term. In practical terms, these two lines of evidence can be integrated: if resistance and conditioning can improve velocity capacity [11,39], then enhanced strength and power may also contribute to greater “fatigue resistance” of throwing performance, although this hypothesis requires direct testing using standardized fatigue models and repeated performance assessments.

### 4.4. Clinical and Practical Relevance of Effect Sizes

Interpreting standardized effects in the applied setting requires caution because the same standardized magnitude can correspond to different absolute changes depending on measurement reliability and between-participant variability. Nevertheless, a small-to-moderate standardized decline in velocity may not be trivial in high-level competition, particularly for jump-shot execution where effects appear more consistent. For accuracy, the uncertainty means practitioners should avoid overgeneralizing a single pooled estimate. A more appropriate applied conclusion is that accuracy may degrade when fatigue is sufficiently high and when accuracy is measured as spatial error or outcome success under constrained tasks, but the magnitude is not yet reliably quantifiable across contexts. This has practical implications: coaches may consider monitoring velocity as a more robust indicator of fatigue-related decrement in throwing performance, while treating accuracy outcomes as more context- and metric-dependent until measurement approaches are standardized.

### 4.5. Practical Implications for Coaches

From an applied perspective, the findings suggest that coaches should pay particular attention to throwing velocity during fatigue-inducing training drills, especially when players perform jump shots or repeated upper-limb dominant actions. Velocity appears to be the more consistently affected outcome and may therefore be a useful practical marker of fatigue-related performance decline. Coaches may consider incorporating repeated throwing drills under controlled fatigue, monitoring whether players can maintain ball speed across sets, and allowing sufficient recovery when velocity reductions become evident.

Accuracy/precision should also be monitored, but current evidence suggests that it is more difficult to interpret because different testing protocols capture different aspects of performance. Therefore, coaches should use consistent target tasks and scoring systems within their own training environment rather than comparing accuracy values across different protocols. Practically, fatigue-resistant shooting drills should combine match-like movement, decision-making, and repeated throwing actions, while avoiding excessive upper-limb fatigue that may compromise throwing mechanics.

### 4.6. Limitations and Implications for Generalizability

Several limitations affect how confidently these findings can be generalized. First, the evidence base is dominated by non-randomized repeated-measures designs and small samples, which increases vulnerability to confounding (e.g., prior training load, recovery, learning effects, motivation, and pacing) and reduces precision of effect estimates. Second, the operationalization of fatigue differed substantially across studies (localized vs. whole-body vs. match simulation; varying duration, intensity, verification), meaning that “fatigue” is not a single exposure and pooled estimates inevitably average across distinct physiological states. Third, outcome measurement heterogeneity—especially for accuracy—was a major constraint. Without consensus on accuracy definitions (e.g., spatial error vs. success rate vs. points) and without consistent reporting of reliability and minimal detectable change, it is difficult to distinguish true performance impairments from metric-specific noise. Fourth, some studies reported outcomes derived from multiple throws per participant, raising unit-of-analysis concerns if repeated throws were treated as independent observations rather than clustered within players; this could lead to overly narrow confidence intervals and overestimated precision. Finally, generalizability is constrained by sample characteristics: many studies used single-team samples and limited representation across sex, age groups, and playing positions, which are plausible effect modifiers in throwing performance and fatigue responses. Additionally, restricting inclusion to peer-reviewed English-language articles may have introduced language and publication bias. Although SPORTDiscus is a relevant database for sports science research, it was not searched because institutional access was unavailable; therefore, some relevant records indexed exclusively in SPORTDiscus may have been missed. However, the use of multiple major databases, Google Scholar, and backward and forward citation tracking likely reduced this risk. Another limitation was the limited reporting of reliability data for the throwing-performance protocols. Although velocity outcomes were usually obtained using objective measurement methods, protocol-specific reliability was not consistently reported. For accuracy outcomes, reliability was particularly difficult to evaluate because of the diversity of metrics and scoring systems used across studies. This lack of standardized reliability reporting limits interpretation of whether observed changes exceeded measurement error.

### 4.7. Future Directions

Future research would benefit most from improved standardization and reporting. Accuracy outcomes in particular require greater harmonization, either through a core outcome set or at minimum through a clear taxonomy separating “success-based” accuracy (hits/goals) from “error-based” accuracy (distance/deviation), alongside consistent reporting of measurement reliability [10]. Studies should report the within-participant pre–post correlation or change-score variability to support correct paired-effect estimation in meta-analysis and should pre-register primary outcomes and analysis plans to reduce selective reporting. Fatigue models should be described and verified consistently (e.g., HR, lactate, RPE, and/or performance decrement criteria) and should prioritize ecologically valid match-like demands when the goal is applied inference. Analytically, repeated throws should be handled using participant-level aggregation or multilevel modeling to respect clustering, and future trials should purposely test effect modifiers such as throwing task (jump vs. standing), fatigue type (localized vs. whole-body vs. match simulation), sex, age, and playing position. In addition, future studies should report protocol-specific reliability indices for throwing velocity and accuracy outcomes, including intraclass correlation coefficients, coefficients of variation, standard error of measurement, and minimal detectable change. This would improve interpretation of whether fatigue-related changes represent true performance decrements rather than measurement variability.

## 5. Conclusions

This systematic review and meta-analysis examined the acute effects of fatigue on handball throwing performance, with a focus on throwing velocity and accuracy. The findings suggest that fatigue is generally associated with a small-to-moderate reduction in throwing velocity, with subgroup analyses suggesting more consistent effects in jump-shot tasks and under localized upper-limb fatigue conditions. These results suggest that fatigue may compromise ball release speed in competitive contexts, although the magnitude of impairment varies substantially across fatigue protocols and task demands.

For throwing accuracy/precision, the available evidence is insufficient to support a robust pooled conclusion. Although some studies suggest that accuracy may deteriorate under intensive or sport-specific fatigue conditions, the very high heterogeneity and inconsistent outcome definitions mean that the pooled estimate should be interpreted with considerable caution. At present, no uniform effect of fatigue on throwing accuracy/precision can be established across handball populations and testing protocols.

## Figures and Tables

**Table 1:** Characteristics, results, and effect sizes of included studies (n = 10).

Study	Design/Setting	Participants	Fatigue Protocol	Throwing Task & Outcomes	Results (Pre vs. Post/Condition A vs. B)	Effect Size (Hedges’ g, 95% CI)
Akyüz et al., 2019 [35]	Pre–post experimental	16 elite male players	30–15 Intermittent Fitness Test (30–15 IFT) to fatigue/failure (whole-body intermittent field fatigue)	Overarm throws to targets; outcomes: accuracy (hits) and ball speed (km/h) for accurate shots	Accuracy (hits): 5.31 ± 2.08 → 5.87 ± 2.06; Ball speed (accurate shots): 66.75 ± 5.83 → 67.02 ± 4.66	Accuracy: g = +0.42 (−0.50 to 1.34); Ball speed: g = +0.06 (−0.88 to 1.00)
Andrade et al., 2016 [13]	Pre–post experimental	10 male players	Simulated game activities (SGA)	Standing & jump throws; outcome: ball velocity (m/s)	Standing velocity: 23.6 ± 3.1 → 23.2 ± 3.2; Jump velocity: 22.8 ± 2.5 → 22.5 ± 3.2	Standing: g = −0.17 (−0.82 to 0.48). Primary-analysis selection: standing throw (one comparison per study per outcome)
Bauer et al., 2020 [14]	Pre–post experimental	24 male adolescent players	Repeated metronome-paced push-up sets to upper-body fatigue/failure (60 beats·min^−1^; termination when <60% of initial maximum repetitions could be completed)	Standing throw from 7 m; outcome: throwing velocity (km/h)	84.5 ± 7.2 → 82.3 ± 6.6	Velocity: g = −0.40 (−0.73 to −0.07)
Belčić et al., 2021 [26]	Pre–post experimental	10 elite players (46 analyzed shots pre + 46 post)	30–15 intermittent fitness test to failure (high lactate)	Jump shots; outcomes: ball speed (km/h) and accuracy error (cm)	Ball speed: 87.28 ± 5.47 → 84.52 ± 5.89; Accuracy error: 36.20 ± 25.71 → 33.17 ± 27.30	Ball speed: g = −0.43 (−0.72 to −0.15); Accuracy (inverted so higher = better): g = +0.08 (−0.22 to 0.38)
Moss & Twist, 2015 [34]	Randomized crossover/simulated match-play	8 youth male players	Two simulated match-play work–rest conditions (LONG vs. SHORT)	Throwing velocity during simulated play	Throwing velocity: SHORT 70.02 ± 7.40 vs. LONG 69.04 ± 5.57; throwing velocity was slightly higher in SHORT, while SHORT was also associated with lower physiological load and better preserved performance overall	Velocity (LONG vs. SHORT): g = −0.12 (−0.80 to 0.56)
Plummer & Oliver, 2015 [36]	Pre–post experimental	10 male players	Progressive treadmill protocol to exhaustion	Jump shot; outcomes: ball speed (mph) and accuracy (%)	Ball speed: 41.96 ± 3.02 → 41.96 ± 3.04; Accuracy: 54.49 ± 13.15 → 60.75 ± 13.94	Ball speed: g = 0.00 (−0.66 to 0.66); Accuracy: g = +0.48 (−0.17 to 1.13)
Plummer & Oliver, 2017 [37]	Pre–post experimental	11 male players	Localized upper-limb fatigue (medicine ball throws)	Jump shot; outcomes: ball speed (m/s) and accuracy (%)	Ball speed: 19.8 ± 2.0 → 18.8 ± 2.1; Accuracy: 60.8 ± 14.1 → 52.8 ± 12.7	Ball speed: g = −0.53 (−1.00 to −0.06); Accuracy: g = −0.50 (−0.97 to −0.03)
Torabi et al., 2025 [33]	Pre–post experimental, 2-group comparison	30 female players (15 pain/15 no pain)	Functional repeated-throwing fatigue protocol: 6 rounds of 10 standing throws (first 5 at 75–85% of maximal pre-fatigue velocity, next 5 at 90–100%), with 1 min recovery between rounds; terminated earlier if Borg CR-10 = 10	Standing throw; outcome: throwing velocity (km/h)	Pain group: 72.5 ± 5.5 → 73.9 ± 6.5; No-pain group: 72.4 ± 5.4 → 74.0 ± 5.2	Pain group: g = +0.29 (−0.38 to 0.96); No-pain group: g = +0.33 (−0.35 to 1.00)
Zapartidis et al., 2007 [32]	Repeated measures across simulated game stages	16 female players	Simulated game activities across blocks (IM → B3)	Throws; outcomes: ball velocity (m/s) and accuracy deviation (cm)	Velocity: 16.22 ± 1.47 (IM) → 16.60 ± 1.59 (B3); Deviation: 20.33 ± 5.49 → 33.14 ± 7.33 (worse)	Velocity: g = +0.19 (−0.61 to 0.99); Accuracy (inverted so higher = better): g = −1.20 (−2.02 to −0.38)
Nuño et al., 2016 [15]	Repeated measures across progressive fatigue circuits	20 male players	Handball-specific circuit ×4 (progressively reduced recovery)	7 m throws to customized target; outcomes: release velocity and accuracy points	Accuracy points: 50.23 ± 6.49 (pre) → 13.77 ± 6.92 (after circuit 4); Velocity: 23.75 ± 1.02 → 22.10 ± 0.79	Accuracy: g = −4.05 (−4.76 to −3.34); Velocity: g = −1.60 (−2.06 to −1.14)

Abbreviations: CI, confidence interval; CR-10, category-ratio 10 scale; g, Hedges’ g; HR, heart rate; km/h, kilometers per hour; m/s, meters per second; mph, miles per hour; RPE, rating of perceived exertion; SGA, simulated game activity; 30–15 IFT, 30–15 Intermittent Fitness Test. Notes: Values are presented as mean ± standard deviation unless otherwise stated. Effect sizes are reported as Hedges’ g with 95% confidence intervals and were oriented so that negative values indicate worse performance under fatigue. Accuracy outcomes based on error or deviation were inverted where necessary so that higher values consistently reflected better performance. When multiple eligible conditions or time points were reported within a study, a single comparison was selected for meta-analysis based on predefined criteria. In Belčić et al. [26], summary statistics are reported at the throw level (number of analyzed shots) rather than as participant-level summaries, which may overstate precision. Torabi et al. [33] reported subgroup-specific data (pain and no-pain groups); for the primary meta-analysis, a single combined comparison was used to avoid double counting the study. The symbol “→” indicates the change from pre-fatigue to post-fatigue values (or between experimental conditions).

**Table 2:** Fatigue protocols used to induce fatigue prior to (or during) throwing-performance testing in the included studies.

Study (Author, Year)	Fatigue Type and Setting	Fatigue Protocol	Intensity/Termination Criterion	Fatigue Verification/Monitoring and Timing of Post-Fatigue Throwing Assessment
Akyüz et al., 2019 [35]	Whole-body, running-based intermittent fatigue (field)	Fatigue was induced using the 30–15 Intermittent Fitness Test (30–15 IFT), consisting of 30 s shuttle runs interspersed with 15 s walking recovery over a 40 m course paced by audio beeps. Initial speed was individualized at 75% of vVO_2_max and increased by 0.5 km·h^−1^ every 45 s stage. Participants were required to reach predefined 3 m zones at each beep and walked to the nearest line during recovery to begin the next stage.	Exhaustion was defined as the inability to match the required distance according to the audio signal on three consecutive occasions, or until the participant reached 90% of maximal heart rate.	Heart rate was monitored during the 30–15 IFT, with the protocol ending when the predefined exhaustion criterion was met. After completion of the fatigue protocol, participants repeated the same shooting-performance procedure used at baseline.
Andrade et al., 2016 [13]	Whole-body, handball-specific simulated activity (field/court)	Fatigue was induced using simulated game activities based on match observations, requiring each participant to complete approximately 100 steps and 20 goal-directed throws, reflecting the average locomotor and throwing demands recorded in the final three matches.	The protocol consisted of a single simulated bout reflecting match load, rather than a progressive test to failure.	Heart rate was monitored during the simulated activities (mean ≈153 bpm, ~77% of predicted HRmax). Post-fatigue throwing was assessed after the simulated game following the study protocol.
Bauer et al., 2020 [14]	Local/upper-body fatigue (laboratory/court)	Fatigue was induced using metronome-paced push-ups at 60 beats·min^−1^ (2 s per repetition). After establishing each participant’s maximal repetitions, repeated push-up sets were performed at the same cadence with 1 min rest between sets until the participant could no longer complete at least 60% of the initial maximum repetitions.	Fatigue was reached when participants could not maintain at least 60% of their initial maximal repetitions in a set.	The inability to complete at least 60% of the initial maximum repetitions was used as the exhaustion criterion. Throwing was assessed immediately after the final failed set, with approximately 10 s between termination of the push-up protocol and the post-fatigue throw.
Belčić et al., 2021 [26]	Whole-body intermittent fatigue with lactate elevation (court)	Fatigue was induced using a progressive 30–15 intermittent running protocol (30 s running, 15 s recovery) performed to failure. After a standardized warm-up, participants completed 5 baseline jump shots (30 s between shots) aiming at the top corner, underwent the fatigue protocol with blood lactate assessment, and then repeated the same 5 jump shots immediately post-fatigue.	The test was stopped when participants failed to reach the 3 m zone three consecutive times.	Lactate and heart rate were measured (with repeated lactate samples pre/post), and perceived exertion was recorded on a 0–10 scale after the fatigue protocol. Throwing performance was assessed immediately post-fatigue using the same baseline shot procedure.
Moss & Twist, 2015 [34]	Whole-body intermittent match-simulation with handball-specific actions (court)	Fatigue was induced using an intermittent team-sport simulation composed of repeated ~50 s movement circuits with ~10 s rest (≈1 circuit·min^−1^) following a standardized warm-up. Two work–rest distributions were compared: a LONG condition (3 × 13 min work with 2 × 8 min rest) and a SHORT condition (5 × ~7.5 min work with 4 × ~3.75 min rest). In both conditions, handball-specific actions were inserted at fixed time points, including 9 jump shots and 20 moderate contact pushes against a bump pad.	The protocol was time-based rather than to volitional failure, with fatigue induced by accumulated intermittent running and sport-specific actions across scheduled work blocks.	Heart rate was monitored throughout, with blood lactate/glucose and session RPE recorded after completion. Throwing performance was measured repeatedly, using mean velocity from early, middle, and late shot sets per condition with a maximal jump-shot procedure.
Plummer & Oliver, 2015 [36]	Whole-body aerobic fatigue (treadmill)	Fatigue was induced using a constant-load treadmill run to exhaustion set from prior VO_2_max testing. An individualized workload corresponding to ~80% of maximal heart rate was identified from the VO_2_max test, and participants then ran at this intensity until volitional exhaustion.	Termination occurred at volitional exhaustion during constant-load treadmill running at an individualized intensity of ~80% HRmax.	Heart rate was recorded throughout, and perceived exertion was assessed repeatedly using Borg-type ratings. Valid jump shots (meeting preset criteria) were performed immediately after the treadmill fatigue protocol.
Plummer & Oliver, 2017 [37]	Localised upper-extremity fatigue (throwing-arm specific)	Fatigue was induced using a throwing-arm–specific medicine-ball rebounder protocol. Participants performed maximal 2.2 kg medicine-ball throws into a rebounder from 6.10 m approximately every 5 s, from a standardized kneeling position, until volitional fatigue. RPE was recorded every 20 throws.	Fatigue was reached when participants reported they could no longer continue throwing (volitional fatigue).	RPE was used to monitor fatigue progression. Immediately post-fatigue, participants performed valid jump-shot trials for biomechanical and performance analysis.
Torabi et al., 2025 [33]	Functional, handball-specific throwing fatigue (repeated submaximal and maximal throws)	After five maximal standing throws, a functional fatigue protocol (FFP) was performed consisting of six rounds of 10 throws: the first five at 75–85% of pre-calculated maximal throwing velocity and the next five at maximal effort (90–100%). Recovery between rounds was 1 min; within-round recovery was self-paced but <20 s.	The protocol continued for up to 60 throws and was terminated earlier if the participant reported 10/10 (maximal fatigue) on the Borg CR-10 scale.	Rate of perceived exertion was recorded after each set of 10 throws using the Borg CR-10 scale. The post-fatigue maximal throws were captured immediately after the final throw of the FFP.
Zapartidis et al., 2007 [32]	Whole-body handball-specific simulated game activities (court)	Fatigue was induced using a match-derived simulated game activities (SGA) protocol structured as two halves separated by 12 min, each half comprising three 10-min periods with a 1 min timeout in the third period. Each period consisted of repeated ~1 min circuits combining 15 m walking, wall passes with movement, 15 m slow running, a defensive triangle with lateral shuffles and a strength/power action, followed by 15 m fast running or sprinting. Maximal 15 m sprints were inserted every 4th and 8th repetition, jumping actions every 3rd pass, and defensive push-ups were performed each repetition with a vertical block jump every 3rd repetition.	Fatigue was induced through repeated sport-specific circuits across fixed match-style periods, rather than a single test to exhaustion.	Throwing was embedded within the simulated game activity (SGA): 21 throws total—3 pre-SGA and 3 at the end of each 10-min period across six periods—allowing repeated tracking of throwing changes during progressive match fatigue.
Nuño et al., 2016 [15]	Whole-body handball-specific circuit fatigue with progressive recovery reduction (court)	Fatigue was induced using a handball-specific circuit of four sets of eight laps performed in a 12 × 12 m area, with progressively shorter recovery between laps (80, 40, 20, and 10 s for sets 1–4) and 3 min rest between sets. Each lap included 10 push-ups, a 12 m dribble run, a 15 m run with long/short passes to a teammate, 6 m defensive runs with forward–backward movements, a 6 m shuttle with four direction changes touching cones, and a diagonal 15 m sprint.	The protocol increased difficulty by systematically reducing recovery time, inducing greater central and peripheral fatigue across sets.	Heart rate was recorded, RPE was taken after each set, and blood lactate was sampled at key points (end of set 2 and end of test). After each set, participants performed nine maximal 7-m throws to a custom goal, with accuracy demands in early throws and radar-measured velocity, to track fatigue effects across progressively harder stages.

Abbreviations: 30–15 IFT, 30–15 Intermittent Fitness Test; bpm, beats per minute; CR-10, Borg category-ratio 10 scale; FFP, functional fatigue protocol; HR, heart rate; HRmax, maximal heart rate; RPE, rating of perceived exertion; SGA, simulated game activity; vVO_2_max, velocity at maximal oxygen uptake; VO_2_max, maximal oxygen uptake. Notes: Fatigue verification methods included physiological (heart rate, blood lactate), perceptual (RPE), and performance-based criteria. Post-fatigue throwing assessments were performed immediately following protocol completion unless otherwise specified.

**Table 3:** Domain-level risk-of-bias judgments for the included studies. Section (**A**) presents the nine non-randomized studies assessed with ROBINS-I, and Section (**B**) presents the randomized crossover study assessed with RoB 2.

**(A) Non-randomized studies assessed with ROBINS-I.**						
**Study**	**Confounding**	**Selection of** ** Participants**	**Deviations from Intended** ** Interventions**	**Missing** ** Data**	**Measurement of** ** Outcomes**	**Selection of Reported** ** Result**
Plummer & Oliver, 2015 [36]	Low	Low	Moderate	Low	Moderate	Moderate
Akyüz et al., 2019 [35]	Low	Low	Moderate	Low	Moderate	Moderate
Andrade et al., 2016 [13]	Low	Low	Low	Low	Low	Low
Bauer et al., 2020 [14]	Low	Low	Moderate	Low	Low	Low
Belčić et al., 2021 [26]	Low	Low	Low	Low	Low	Moderate
Plummer & Oliver, 2017 [37]	Low	Low	Low	Low	Moderate	Moderate
Torabi et al., 2025 [33]	Serious	Low	Low	Low	Moderate	Moderate
Zapartidis et al., 2007 [32]	Moderate	Low	Moderate	Low	Low	Moderate
Nuño et al., 2016 [15]	Serious	Low	Low	Low	Moderate	Serious
**(B) Randomized crossover study assessed with RoB 2.**						
**Study**	**Randomization** ** Process**	**Carryover** ** Effects**	**Deviations from Intended** ** Interventions**	**Missing Outcome** ** Data**	**Measurement of** ** Outcomes**	**Selection of Reported** ** Result**
Moss & Twist, 2015 [34]	Low	Some concerns	Low	Low	Low	Some concerns

Abbreviations: RoB 2, revised Cochrane risk-of-bias tool for randomized trials; ROBINS-I, Risk Of Bias In Non-randomized Studies—of Interventions.

## Data Availability

No new data were created in this study. Data supporting the reported results are available within the article and its Supplementary Materials.

## Supplementary Materials

The following supporting information can be downloaded at: https://www.mdpi.com/article/10.3390/jfmk11020191/s1. Supplementary File S1: PRISMA 2020 checklist; Supplementary File S2: Extracted dataset and effect-size computation data used for the meta-analysis.

## Abbreviations

The following abbreviations are used in this manuscript:

CI	confidence interval
CR-10	category-ratio 10 scale
FFP	functional fatigue protocol
GRADE	Grading of Recommendations Assessment, Development and Evaluation
HR	heart rate
HRmax	maximal heart rate
PRISMA	Preferred Reporting Items for Systematic Reviews and Meta-Analyses
PROSPERO	International Prospective Register of Systematic Reviews
RPE	rating of perceived exertion
RoB 2	Risk of Bias 2 tool
ROBINS-I	Risk Of Bias In Non-randomized Studies—of Interventions
SGA	simulated game activity
SMD	standardized mean difference
VO_2_max	maximal oxygen uptake
vVO_2_max	velocity at maximal oxygen uptake
PECOS	Participants, Exposure, Comparator, Outcomes, Study design
